# Editorial: Narrating the environment: innovation, looks and stories on real and virtual boundaries

**DOI:** 10.3389/fpsyg.2026.1879399

**Published:** 2026-06-25

**Authors:** Eugenio De Gregorio, Lavinia Cicero, Federica Caffaro, Lorenza Tiberio, Erica Löfström

**Affiliations:** 1 Università Link Campus, Rome, Italy; 2 Universitas Mercatorum, Rome, Italy; 3 Universita degli Studi Roma Tre, Rome, Italy; 4 NTNU—Norvegian University of Science and Technology, Trondheim, Norway

**Keywords:** environmental psychology, explainable artificial intelligence, innovative methodologies, narrative ecologies, place attachment, pro-environmental behavior, smart technologies, sustainable sciences

The Research Topic offers a timely and multifaceted contribution to environmental psychology by bringing together a set of works that interrogate how environments are perceived, constructed, mediated, and acted upon. Across empirical, methodological, and conceptual contributions, a common thread emerges: the environment is not merely a physical substrate but can be understood as a narrative field—continuously shaped by symbolic, technological, and psychological processes. The articles collected here collectively advance understanding of environmental experience as a dynamic interplay among representation and action, materiality and mediation, and real and virtual domains.

This editorial aims to synthesize and critically discuss the contributions in this Research Topic, highlighting their innovative aspects and situating them within broader theoretical and methodological developments. Rather than treating the articles as discrete contributions, we approach them as components of a broader intellectual movement, which increasingly reframes environmental psychology through the lenses of narrative, complexity, and interdisciplinarity.

## Mediating the environment: from information to action

A significant portion of the contributions addresses the mechanisms through which environmental awareness is translated into pro-environmental behavior (PEB), with particular attention to the mediating role of communication systems, cognitive processes, and institutional structures. These works collectively challenge linear models of behavior change and instead foreground the complexity of psychological and contextual factors that shape environmental action.

Liao provides a compelling starting point by integrating Media System Dependency Theory (MSDT) with Norm Activation Theory (NAT), thereby bridging two traditionally separate domains: media studies and environmental psychology. The study demonstrates that media exposure, attention, dependency, and credibility influence pro-environmental behavior indirectly, through their impact on awareness of consequences and ascription of responsibility. Crucially, the findings nuance the conventional assumptions of NAT by showing that these components do not exert consistent direct effects on behavior. Instead, their influence is mediated by personal norms and moderated by self-efficacy, suggesting that the relationship between information and action is contingent, layered, and context-dependent. Methodologically, the combination of F-DEMATEL and structural equation modeling allows the study to capture both causal structures and empirical relationships, offering a robust model for future research.

The emphasis on mediation and internalization is further developed by Molkenthin et al., who adopt Organismic Integration Theory to explore how different forms of motivation influence the adoption and diffusion of PEB. Their findings underscore the importance of internalized regulation, demonstrating that behaviors aligned with personal values and identity are not only more frequent but also more likely to extend across different domains of everyday life. This perspective reframes pro-environmental behavior as a systemic phenomenon, one that spreads through individuals' behavioral repertoires rather than remaining confined to isolated actions. In doing so, the study challenges the predominance of external incentives and highlights the transformative potential of intrinsic motivation.

Wu et al. contribute to this line of inquiry by examining participation in personal transportation carbon trading through an environment–cognition–intention framework. By combining structural equation modeling with fuzzy-set qualitative comparative analysis (fsQCA), the authors reveal that participation intention emerges from multiple configurational pathways rather than a single causal chain. The interplay between social norms, perceived usefulness, self-efficacy, and perceived risk illustrates the complexity of behavioral decision-making in environmental contexts. This configurational approach resonates with Zhang et al., who apply a Drive–State–Pressure model to employees' green behavior in the hospitality sector. Their findings show that institutional pressures and individual agency interact in nuanced ways, producing distinct patterns of task-oriented and voluntary green behaviors.

Taken together, these studies signal a shift away from reductionist models of environmental behavior toward more integrative and dynamic frameworks. They suggest that pro-environmental action is not simply the outcome of attitudes or knowledge but the product of complex interactions among media systems, cognitive processes, motivational structures, and institutional contexts. Innovation, in this sense, lies not only in the introduction of new variables but in the reconfiguration of the conceptual landscape itself, moving toward a more holistic understanding of behavior as situated, mediated, and emergent.

## Aesthetic and immersive mediations: rethinking the boundaries of nature

A second group of contributions explores the perceptual and affective dimensions of environmental experience, with a particular focus on the role of aesthetic and immersive mediations. These studies challenge the traditional distinction between real and simulated environments, demonstrating that representations of nature can, under certain conditions, evoke responses comparable to those elicited by direct exposure.

Chen et al. provide empirical evidence for this claim through a study that combines virtual reality (VR) and electroencephalography (EEG) to investigate the restorative effects of nature-themed artworks. Their results show that such artworks can produce physiological and psychological benefits similar to those associated with natural elements, including reductions in blood pressure and increases in alpha wave activity. This finding has profound implications for environmental design, suggesting that symbolic representations of nature can function as effective substitutes in contexts where direct access to natural environments is limited. The use of VR and EEG not only enhances the methodological rigor of the study but also exemplifies the potential of integrating technological tools into environmental psychology.

The theme of immersive mediation is further developed by Godley et al., who examine the impact of a multisensory art installation on student wellbeing in a university setting. Their findings indicate significant improvements in mood and reductions in stress, highlighting the therapeutic potential of immersive art experiences. Importantly, the study combines quantitative measures with qualitative insights, capturing both measurable outcomes and subjective experiences. This dual approach reinforces the idea that environmental interventions operate not only at the level of physiology but also through processes of meaning-making and interpretation.

Löfström et al. extend the discussion by introducing the concept of disruptive eco-visualization. Rather than aiming solely to inform or represent, these interventions are designed to provoke emotional and cognitive responses that challenge anthropocentric perspectives and stimulate reflection on human–nature relationships. The workshops described in the study reveal both the potential and the ambivalence of such approaches, as participants experience reconnection with nature alongside feelings of anxiety or skepticism. This tension underscores the complexity of environmental narratives and the need to balance emotional engagement with critical reflection.

Collectively, these contributions suggest that the boundaries between real and virtual environments appear increasingly porous, and that aesthetic and immersive mediations play a central role in shaping environmental experience. They highlight the capacity of art, design, and technology to function as both representational and transformative tools, capable of influencing perception, emotion, and behavior in profound ways.

## Constructing place: identity, attachment, and narrative space

The relationship between individuals and environments is further explored through studies that focus on place, identity, and narrative construction. These works emphasize that environments are not merely experienced but actively constructed through interactions that generate meanings, attachments, and identities.

Zhang investigates the role of recreational landscape perception in shaping pro-environmental behavior, demonstrating that place identity and behavioral spillover mediate this relationship. By integrating natural, cultural, and experiential dimensions into a unified framework, the study shows how localized experiences can influence broader patterns of behavior. This perspective highlights the importance of emotional and symbolic connections to place, suggesting that sustainable behavior is rooted not only in cognitive evaluations but also in affective bonds.

A similar emphasis on emotional and symbolic dimensions is found in Li and Leng, who examine the role of emotional restoration resources in fostering sustainable rural development. Their systems psychology approach conceptualizes place attachment as a dynamic process that links emotional experiences to behavioral outcomes. The notion of “emotional infrastructures” introduced in the study represents a significant conceptual innovation, expanding the understanding of infrastructure beyond its physical components to include psychological and social dimensions.

Ropert et al. offer a methodological and epistemological contribution by proposing a framework for mapping place-assemblages. Drawing on critical cartography and environmental psychology, the authors argue that maps are not neutral representations but narrative devices that produce and organize spatial meanings. Their approach integrates qualitative and visual methods to capture the complex interplay between material, symbolic, and political dimensions of place. This perspective challenges traditional positivist approaches and opens new avenues for exploring the narrative construction of environments.

Brown complements these empirical and methodological contributions with a conceptual analysis that reframes the built environment as a system of reified concepts organized within a Cartesian grid. By emphasizing the role of design in shaping perception and behavior, the paper aligns with theories of distributed cognition and the extended mind. It suggests that environments are not passive settings but active participants in cognitive processes, guiding perception and action through their structured organization.

Together, these studies underscore the importance of narrative and symbolic processes in the construction of environmental experience. They reveal that places are not fixed entities but dynamic assemblages shaped by interactions, representations, and power relations. Innovation in this domain lies in the integration of affective, cognitive, and political dimensions, as well as in the development of methods capable of capturing their complexity.

## Data, design, and the emergence of intelligent environments

The final group of contributions addresses the role of technological and methodological innovation in the study and design of environments. These works highlight the potential of data-driven approaches and interdisciplinary frameworks to enhance our understanding of environmental behavior and perception.

Izmir Tunahan et al. exemplify this trend through their use of passive infrared sensors, machine learning, and explainable AI to analyze seating preferences in academic libraries. By modeling occupancy patterns and identifying key factors influencing seat choice, the study provides actionable insights for the design of user-centered environments. The integration of large-scale behavioral data with interpretability tools represents a significant methodological advance, enabling researchers to move beyond descriptive analyses toward predictive and explanatory models.

Wei et al. extend this approach by proposing a design-integrated framework for neuroarchitectural research. Their multi-phase methodology, which combines image-based evaluations, object-based interactions, and immersive experiences, aims to bridge the gap between empirical rigor and ecological validity. By positioning architectural environments as active generators of perceptual and behavioral data, the framework challenges traditional distinctions between research and design, suggesting a more iterative and collaborative approach.

These contributions point toward the emergence of intelligent environments that are responsive to human needs and behaviors. They demonstrate how technological tools can enhance both the analysis and the design of spaces, fostering environments that support wellbeing, productivity, and sustainability. At the same time, they raise important questions about the role of technology in mediating environmental experience, highlighting the need for critical reflection on issues of privacy, agency, and authenticity.

## Toward a narrative ecology of environment

The contributions to this Research Topic collectively point toward a reconceptualization of environmental psychology as a narrative ecology. In this emerging paradigm, environments are understood as complex systems of meanings, practices, and representations through which individuals and groups interpret, negotiate, and act upon their surroundings.

Three overarching themes can be identified. First, the environment is increasingly conceptualized as a narrative construct, shaped by media, art, design, and social interactions. Second, there is a growing recognition of complexity, reflected in the adoption of configurational and integrative approaches that capture the interplay of multiple factors. Third, representation is no longer seen as passive but as an active force that influences perception, emotion, and behavior.

At the same time, the contributions reveal tensions and challenges that warrant further exploration. The increasing reliance on technological mediation raises questions about the nature of environmental experience and the potential risks of detachment from physical reality. Similarly, the focus on individual-level processes must be complemented by greater attention to structural and political dimensions, as highlighted by critical approaches to mapping and place-making.

## Conclusion

The Research Topic “*Narrating the Environment*” demonstrates the richness and diversity of contemporary environmental psychology, offering innovative perspectives that transcend traditional disciplinary boundaries. By integrating insights from psychology, design, architecture, and critical theory, the contributions advance a holistic understanding of environmental experience that encompasses perception, emotion, cognition, and action.

Future research should continue to build on this interdisciplinary momentum, exploring how narrative, technology, and social processes can be harnessed to foster more sustainable and meaningful relationships between humans and their environments. In doing so, environmental psychology can move beyond describing the world as it is, toward actively shaping the environments of the future.

